# Temporal Trends and Forecasted Cardiac Arrest and Hypertension-Related Mortality in U.S. Adults, 2000–2035: A Nationwide CDC WONDER Multiple Cause-of-Death Study

**DOI:** 10.1007/s44197-026-00588-x

**Published:** 2026-05-25

**Authors:** Saifullah Khan, FNU Arsheen, Muneeb Fareed, Tahreem Mari, Muhammad Hussain, Preet Memon, S M Aleem Hussain, Muhammad Hassan, Asma Naz, Yusra Ejaz, Gregg C. Fonarow, Hasibullah Aminpoor

**Affiliations:** 1 Department of Dow Medical College, Dow University of Health Sciences, Karachi, Pakistan; 2 Ahmanson-UCLA Cardiomyopathy Center, Ronald Reagan-UCLA Medical Center, Los Angeles, CA USA; 3 Faculty of Medicine, Kabul University of Medical Sciences “Abu Ali Ibn Sina”, Kabul, Afghanistan

**Keywords:** Cardiac arrest, Hypertension, Mortality trends, Forecasting, Temporal trends, Cardiovascular mortality

## Abstract

**Background:**

Cardiac arrest (CA) occurring in the setting of hypertension (HTN) represents a critical and under-recognized intersection of acute cardiovascular catastrophe and chronic vascular disease in the United States (U.S.). The coexistence of sudden cardiac dysfunction and long-standing HTN may amplify morbidity and mortality and contribute to persistent demographic and geographic disparities. This study characterizes 2000–2024 national trends in CA with concurrent HTN among U.S. adults and projects age-adjusted mortality rates (AAMRs) through 2035.

**Methods:**

We conducted a retrospective analysis of Multiple Cause-of-Death data from the CDC WONDER database for CA with HTN mortality among adults aged ≥ 25 years (2000–2024). We calculated AAMRs and used joinpoint regression to estimate annual percent changes (APCs) and the average APC (AAPC) with 95% confidence intervals (CIs). We projected AAMRs through 2035 using Auto-ARIMA and Prophet time-series models in R (v4.5.0) and evaluated model performance using the root mean squared error (RMSE).

**Results:**

From 2000 to 2024, there were 1,693,840 deaths from CA with concurrent HTN. Overall AAMR rose from 24.56 to 31.68, with an AAPC of 0.82 (*p* < 0.000001). Men had higher AAMRs than women (overall AAMR: men 32.89; women 25.95). Among races, non-Hispanic (NH) Black/African American adults had the highest mortality (AAMR: 58.25). Older adults (≥ 65 years) bore the highest burden (AAMR: 122.60). Geographic differences were observed, with the West having the highest burden (AAMR: 43.75). Urban areas had higher AAMRs than rural areas (28.88 vs. 25.50). Most deaths (43.78%) occurred in medical facilities. Forecasting to 2035 indicates continued rises, particularly among men, NH Black/African American adults, older adults, West and rural areas.

**Conclusion:**

CA with concurrent HTN imposes a substantial and rising mortality burden, with pronounced demographic and geographic disparities. Continued surveillance and targeted public-health interventions are warranted.

**Supplementary Information:**

The online version contains supplementary material available at 10.1007/s44197-026-00588-x.

## Introduction

Cardiac arrest (CA) remains a leading cause of sudden mortality. Out-of-hospital CAs occur on the order of hundreds of thousands annually, and recent registry analyses estimate incidence rates of approximately 50–84 per 100,000, with low overall survival to discharge [1]. Forecasting studies anticipate that cardiovascular morbidity and costs will rise markedly over the coming decades, with broad projections showing cardiovascular disease (CVD) costs and productivity losses increasing substantially by 2050 [2]. The mortality and economic burden of arrest and arrest-related CVD already account for large shares of national spending on heart disease and stroke [3]. Annual public health reports and databases (including CDC WONDER and NCHS products) therefore remain essential for tracking these trends and allocating resources [4]. Hypertension (HTN) affects roughly half of U.S. adults in recent surveillance (≈ 48%) and is responsible for a large, persistent share of population-level cardiovascular risk [4]. Uncontrolled HTN alone is associated with very large annual health-care expenditures—estimated in the low-to-mid hundreds of billions per year in recent U.S. analyses—and contributes substantially to downstream costs from heart disease, stroke, kidney disease, and disability [5].

HTN and CA intersect epidemiologically and clinically. Population and case–control studies report that HTN and its sequelae (left ventricular hypertrophy, ischemic heart disease, myocardial fibrosis) modify the risk and substrate for sudden cardiac arrest, while antihypertensive treatment appears to alter that risk in some cohorts [6]. When CA occurs in patients with HTN there poorer immediate and long-term outcomes, and post-arrest blood-pressure abnormalities—both hypotension and extreme HTN—are linked to higher mortality after return of spontaneous circulation [7]. Common upstream risk factors—atherosclerosis, diabetes, obesity, smoking, and sedentary lifestyle—drive both conditions and increase case fatality.

To address knowledge gaps, we conducted a retrospective CDC WONDER analysis of Multiple Cause-of-Death data (2000–2024) to characterize temporal trends, demographic and geographic disparities, and to forecast age-adjusted mortality rates for CA with concurrent HTN through 2035 to inform targeted prevention and resource allocation.

## Methods

### Study Population and Setting

We analyzed national mortality data to examine trends in cardiac arrest (CA) and hypertension (HTN) using the CDC Wide-Ranging Online Data for Epidemiologic Research (WONDER) Multiple Cause-of-Death (MCD) database from 2000–2024.Mortality cases were defined as deaths in which either cardiac arrest or hypertension appeared as an underlying or contributing cause of death in the CDC WONDER Multiple Cause of Death database. Mortality cases were defined as deaths in which cardiac arrest and/or hypertension were listed as either the underlying cause or a contributing cause of death in the CDC WONDER Multiple Cause-of-Death database, based on ICD-10 coding. CA-related deaths were identified using ICD-10 code ICD-10 code I46, and HTN-related deaths were identified using ICD-10 codes ICD-10 codes I10–I15, consistent with prior epidemiologic studies [8–12]. The study followed STROBE guidelines [13]. Institutional Review Board approval was not required, as all data were publicly available and de-identified.

### Data Extraction

Variables included overall mortality, sex, race/ethnicity (Hispanic/Latino; non-Hispanic [NH] White, Black, Asian/Pacific Islander, American Indian/Alaska Native), U.S. Census region, state, urbanization status (urban ≥ 50,000; rural < 50,000), and age groups: younger adults (25–44), middle aged adults (45–64), and older adults (≥ 65) years. Urban–rural classification followed the 2013 NCHS Urban–Rural Classification Scheme [14]. Place of death was categorized as home, hospice, nursing home/long-term care, medical facility, or other/unknown.

### Statistical Analysis

Age-adjusted mortality rates (AAMRs) per 100,000 population, standardized to the 2000 U.S. population, were calculated to assess temporal trends in CA and HTN mortality [15]. Mean AAMRs over the study period were calculated using the average function in Microsoft Excel. Joinpoint regression (version 5.4.0, National Cancer Institute) was used to identify statistically significant changes in trend segments over time, with permutation tests used to determine the number of joinpoints [16]. Annual percent change (APC) with 95% confidence intervals (CIs) was calculated for each segment, with trends classified as increasing or decreasing if the APC differed significantly from zero (*p* < 0.05).

### Time-Series Forecasting

The Auto-ARIMA model was implemented using the forecast package in R, which automatically selects the optimal autoregressive (p), differencing (d), and moving average (q) parameters by minimizing the Akaike Information Criterion (AIC). Prior to model fitting, stationarity of the time series was assessed, and seasonal differencing and transformations were applied when necessary to ensure model stability.

The Prophet model, developed by Meta Platforms, was implemented using the prophet package in R [17]. Prophet decomposes time series into trend, seasonal, and irregular components within a generalized additive model framework and incorporates piecewise linear trends with automatically detected changepoints, allowing the model to capture potential structural changes in mortality trends over time.

For model development, data from 2000 to 2020 were used as the training set, while 2021–2024 were used as the validation period for model evaluation, consistent with the structure of annual mortality data [10]. Model performance was evaluated using a rolling one-step-ahead forecasting approach, in which the model was iteratively refitted to predict each subsequent year. Predictive accuracy was assessed using root mean squared error (RMSE), and the model with the lowest RMSE was selected as the final forecasting model. The final model was then refitted using the complete dataset (2000–2024) to generate projections for 2025–2035. Forecasts were reported with 95% confidence intervals to quantify statistical uncertainty.

## Results

Between 2000 and 2024, CA associated with HTN accounted for a total of 1,693,840 deaths among adults aged 25 years and older in the United States (Supplemental Table 1). These deaths occurred across various locations in the country, with 43.78% taking place in medical facilities, 30.24% at the decedents’ homes, 20.77% in nursing homes, 1.10% in hospice facilities, and 3.78% at other locations (Supplemental Table 2).

### Annual Trends for CA with HTN Age-Adjusted Mortality Rate (AAMR)

**Fig. 1:**
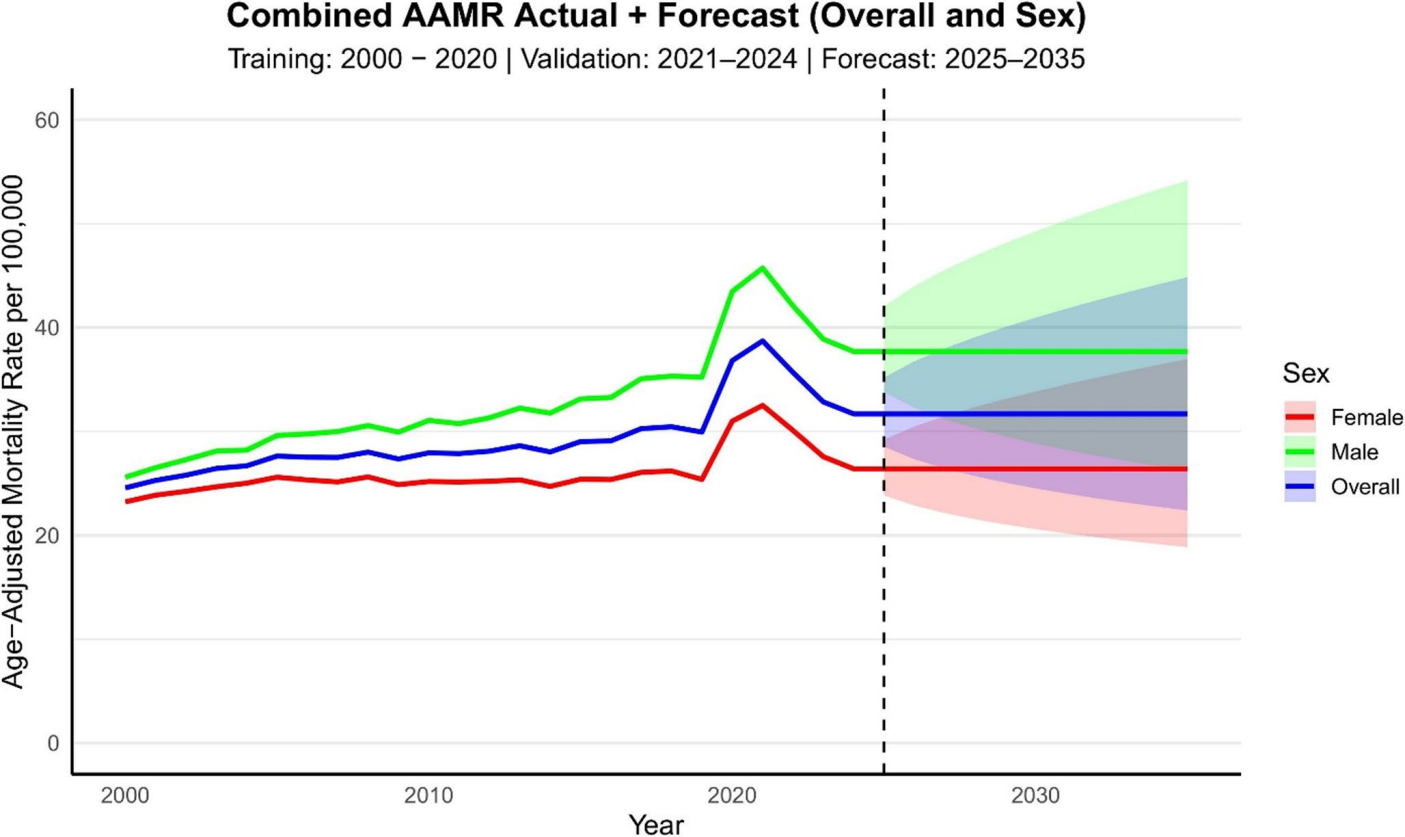
Cardiac arrest and hypertension AAMR Stratified by Overall and Sex per 100,000 Population

The overall AAMR for CA with HTN increased from 24.56 in 2000 to 31.68 in 2024, with stabilization in AAPC of 0.82 (95% CI: 0.53 to 1.04; p value < 0.000001) **(**Table 1**)**. The annual trend for overall AAMR had a steady increase from 2000 to 2018 (APC: 0.84; 95% CI: 0.48 to 1.13; p value = 0.0012), followed by a significantly sharp rise between 2018 and 2021 (APC: 8.98; 95% CI: 5.29 to 10.63; p value < 0.0001). Subsequently, a marked decrease was observed from 2021 to 2024 (APC: −6.82; 95% CI: −9.55 to −4.82; p value < 0.0001). **(**Fig. 1). (Supplemental Table 1).

**Table 1:** AAMR and AAPC for Cardiac arrest and hypertension among U.S. adults aged ≥ 25 years, 2000 to 2024. (*) asterisk sign indicate significant trend

	Trend 1		Trend 2		Trend 3		Trend 4		Trend 5	
	Year	AAPC	Year	APC	Year	APC	Year	APC	Year	APC
Overall	2000–2024	0.83* (0.54 to 1.04)	2000–2018	0.85* (0.49 to 1.14)	2018–2021	8.98* (5.29 to 10.64)	2021–2024	-6.82* (-9.55 to -4.82)	N/A	N/A
*Sex*										
Female	2000–2024	0.28 (-0.03 to 0.49)	2000–2018	0.29 (-0.12 to 0.58)	2018–2021	8.01* (4.00 to 9.73)	2021–2024	-6.91* (-9.95 to -4.72)	N/A	N/A
Male	2000–2024	1.38* (1.11 to 1.60)	2000–2006	2.91* (1.38 to 6.14)	2006–2018	-0.44 (-1.38 to 0.006)	2018–2021	10.40* (6.96 to 12.35)	N/A	N/A
*Race*										
Hispanic	2000–2024	-0.17 (-0.62 to 0.27)	2000–2018	0.00 (-0.76 to 0.54)	2018–2021	10.07* (4.45 to 13.14)	2021–2024	-10.37* (-16.70 to -6.77)	N/A	N/A
NH American Indian or Alaska Native	2000–2024	1.37* (0.61 to 2.16)	2000–2021	3.28* (2.65 to 4.23)	2021–2024	-11.02* (-22.25 to -4.38	N/A	N/A	N/A	N/A
NH Asian or Pacific Islander	2000–2024	-1.54* (-1.91 to -1.19)	2000–2018	-1.79* (-2.34 to -1.34)	2018–2021	9.78* (4.74 to 12.34)	2021–2024	-10.34* (-14.19 to -7.57)	N/A	N/A
NH Black	2000–2024	-0.25 (-0.67 to 0.29)	2000–2004	2.71 (-0.31 to 10.84)	2004–2017	-1.50* (-4.85 to -1.03)	2017–2021	7.54* (4.37 to 11.86)	2021–2024	-8.31* (-12.13 to -4.61)
NH White	2000–2024	1.20* (0.94 to 1.37)	2000–2018	1.17* (0.85 to 1.42)	2018–2021	8.42* (5.01 to 9.78)	2021–2024	-5.38* (-7.81 to -3.61)	N/A	N/A
*Age Group*										
Younger adults (25–44)	2000–2024	3.07* (2.54 to 3.86)	2000–2005	6.27* (3.36 to 18.61)	2005–2018	2.17 (-3.08 to 2.85)	2018–2021	16.92* (10.35 to 20.63)	2021–2024	-10.30* (-14.46 to -7.23)
Middle Aged adults (45–64)	2000–2024	1.84* (1.50 to 2.13)	2000–2018	2.11* (1.66 to 2.49)	2000–2018	12.53* (8.38 to 14.72)	2021–2024	-9.28* (-12.41 to -6.81)	N/A	N/A
Older Adults (65+)	2000–2024	0.60* (0.33 to 0.80)	2000–2018	0.56* (0.21 to 0.84)	2018–2021	7.96* (4.30 to 9.53)	2021–2024	-6.08* (-8.77 to -4.11)	N/A	N/A
*Urbanization*										
Urban	2000–2020	1.44* (0.97 to 1.71)	2000–2018	0.60* (0.13 to 0.92)	2018–2020	9.25* (2.89 to 11.89)	N/A	N/A	N/A	N/A
Rural	2000–2020	2.80* (2.23 to 3.15)	2000–2018	2.02* (1.16 to 2.43)	2018–2020	10.09* (3.05 to 13.57)	N/A	N/A	N/A	N/A
*Census Region*										
Northeast	2000–2024	0.25 (-0.17 to 0.69)	2000–2004	2.93* (0.30 to 9.40)	2004–2018	-0.38* (-3.58 to -0.02)	2018–2021	7.88* (3.34 to 10.58)	2021–2024	-7.40* (-12.80 to -4.36)
Midwest	2000–2024	1.55* (1.24 to 1.79)	2000–2011	0.56 (-1.87 to 1.18)	2011–2018	2.93* (1.22 to 4.76)	2018–2021	9.57* (6.18 to 11.48)	2021–2024	-5.50* (-8.09 to -3.57)
South	2000–2024	0.74* (0.35 to 1.03)	2000–2018	0.82* (0.25 to 1.21)	2018–2021	8.56* (3.82 to 10.71)	2021–2024	-6.98* (-10.86 to -4.31)	N/A	N/A
West	2000–2024	0.97* (0.71 to 1.18)	2000–2018	0.98* (0.64 to 1.26)	2018–2021	9.36* (5.91 to 10.90)	2021–2024	-6.83* (-9.24 to -4.94)	N/A	N/A

(*) asterisk sign indicate significant trend

Forecasting analyses suggested relative stabilization of overall mortality trends through 2035, with the overall AAMR projected to remain near the 2024 observed level at 31.68 (95% CI: 22.53 to 44.18) (Supplemental Table 9).

### CA with HTN-Related AAMR Stratified by Sex

The AAMR for both men and women increased from 2000 to 2024. Among men, the AAMR increased significantly from 25.59 (95% CI: 25.2 to 25.96) in 2000 to 37.68 (95% CI: 37.34 to 38.03) in 2024. Among women, the AAMR rose from 23.21 (95% CI: 22.93 to 23.5) in 2000 to 26.39 (95% CI: 26.14 to 26.65) in 2024.

Men consistently exhibited higher mean AAMRs compared to women (mean AAMR for men: 32.89; 95% CI: 32.52 to 33.26; for women: 25.95; 95% CI: 25.67 to 26.23) throughout the study timeline.

From 2000 to 2024, the AAMR trend for men significantly increased, while it remained stable for women [men: AAPC: 1.38, (95% CI: 1.11 to 1.6; p value < 0.0001); women: AAPC: 0.28, (95% CI: −0.029 to 0.49; p value = 0.074)].

The AAMR trend for women showed a stable pattern from 2000 to 2018 (APC: 0.28; 95% CI: −0.11 to 0.58; p value = 0.14), followed by a sharp rise from 2018 to 2021 (APC: 8.01; 95% CI: 3.99 to 9.72; p value < 0.000001). This was followed by a pronounced decline between 2021 and 2024 (APC: −6.90; 95% CI: −9.94 to −4.71; p value < 0.0001). Similarly, for men the AAMR trend increased from 2000 to 2018 (APC: 1.46; 95% CI: 1.109 to 1.75; p value = 0.0001), followed by a marked increase between 2018 and 2021 (APC: 9.62; 95% CI: 6.20 to 11.24; p value < 0.0001). (Supplemental Table 3).

Forecasting analyses suggested a plateauing of CA with HTN-related mortality trends through 2035. Projected male and female AAMRs remained comparable to 2024 observed estimates, at 37.68 (95% CI: 26.78 to 54.04) and 26.39 (95% CI: 18.84 to 36.21), respectively **(**Fig. 1**) **(Supplemental Table 9).

### CA with HTN-Related AAMR Stratified by Race/Ethnicity

When stratified by race/ethnicity, mean AAMRs were highest among NH Blacks/African Americans (58.25; 95% CI: 57.17 to 59.33), followed by Hispanics (37.81; 95% CI: 36.81 to 38.8), NH Asians/Pacific Islanders (36.73; 95% CI: 35.34 to 38.12), NH American Indians/Alaskan Natives (26.92; 95% CI: 23.83 to 30.04), and NH Whites (24.82; 95% CI: 24.58 to 25.05).

The AAMR increased across Hispanics (33.77 to 36.02), NH American Indians/Alaskan Natives (17.77 to 26.96), and NH Whites (20.33 to 27.98). Whereas, a decline was observed among NH Asians or Pacific Islanders (40.35 to 30.35) and NH Blacks or African Americans (56.42 to 54.84) from 2000 to 2024.

Notably, the AAMR trend increased from 2000 to 2024 among NH American Indians/Alaskan Natives (AAPC: 1.37 (95% CI: 0.606 to 2.163) (p value < 0.0001)and NH Whites (AAPC: 1.198 (95% CI: 0.937 to 1.373) (p value < 0.0001), while it remained stable with a slight decrease among Hispanics (AAPC: −0.166 (95% CI: −0.622 to 0.269;p value < 0.4323) and NH Blacks/African Americans (AAPC: −0.245, (95% CI: −0.674 to 0.29;p value = 0.247); with a notable decline observed among NH Asians/Pacific Islanders (AAPC: −1.537, (95% CI: −1.911 to −1.192p value < 0.0001). (Supplemental Table 5).

Forecasting analyses demonstrated relative stabilization in race/ethnicity-specific AAMRs through 2035. Projected AAMRs remained near 2024 observed levels for Hispanics at 37.52 (95% CI: 31.39 to 44.84), NH American Indians/Alaskan Natives at 26.96 (95% CI: 11.22 to 64.72), NH Asians/Pacific Islanders at 30.35 (95% CI: 18.54 to 49.66), NH Blacks/African Americans at 58.06 (95% CI: 50.56 to 66.69), and NH Whites at 27.98 (95% CI: 20.61 to 37.97) **(**Fig. 3) (Supplemental Table 9).

### CA with HTN-Related AAMR Stratified by Age

Between 2000 and 2024, CA with HTN resulted in 27,639 deaths among younger adults (25–44), 292,276 among middle-aged adults (45–64), and 1,373,925 among older adults (65+). The AAMRs increased across all age groups from 2000 to 2024: younger adults (0.8 to 1.75); middle-aged adults (10.14 to 16.04); older adults (105.98 to 129.79).

Significant differences in mean AAMRs were exhibited among different age groups, with the older adults showing highest mean AAMRs throughout the time period, followed by middle aged adults and younger adults (mean AAMR: older Adults: 122.6, 95% CI: 121.56 to 123.64; middle-aged Adults: 13.59, 95% CI: 13.34 to 13.84; and younger Adults: 1.37, 95% CI: 1.29 to 1.45).

The AAMR trend increased among younger adults and middle-aged adults, while it remained stable in older adults [younger Adults: AAPC: 3.07, (95% CI: 2.53 to 3.86; p value < 0.0001); Middle-aged Adults: AAPC: 1.84, (95% CI: 1.5 to 2.12; p value < 0.0001); and Older Adults: AAPC: 0.59, (95% CI: 0.32 to 0.79; p value < 0.0001)]. (Supplemental Table 4).

**Fig. 2:**
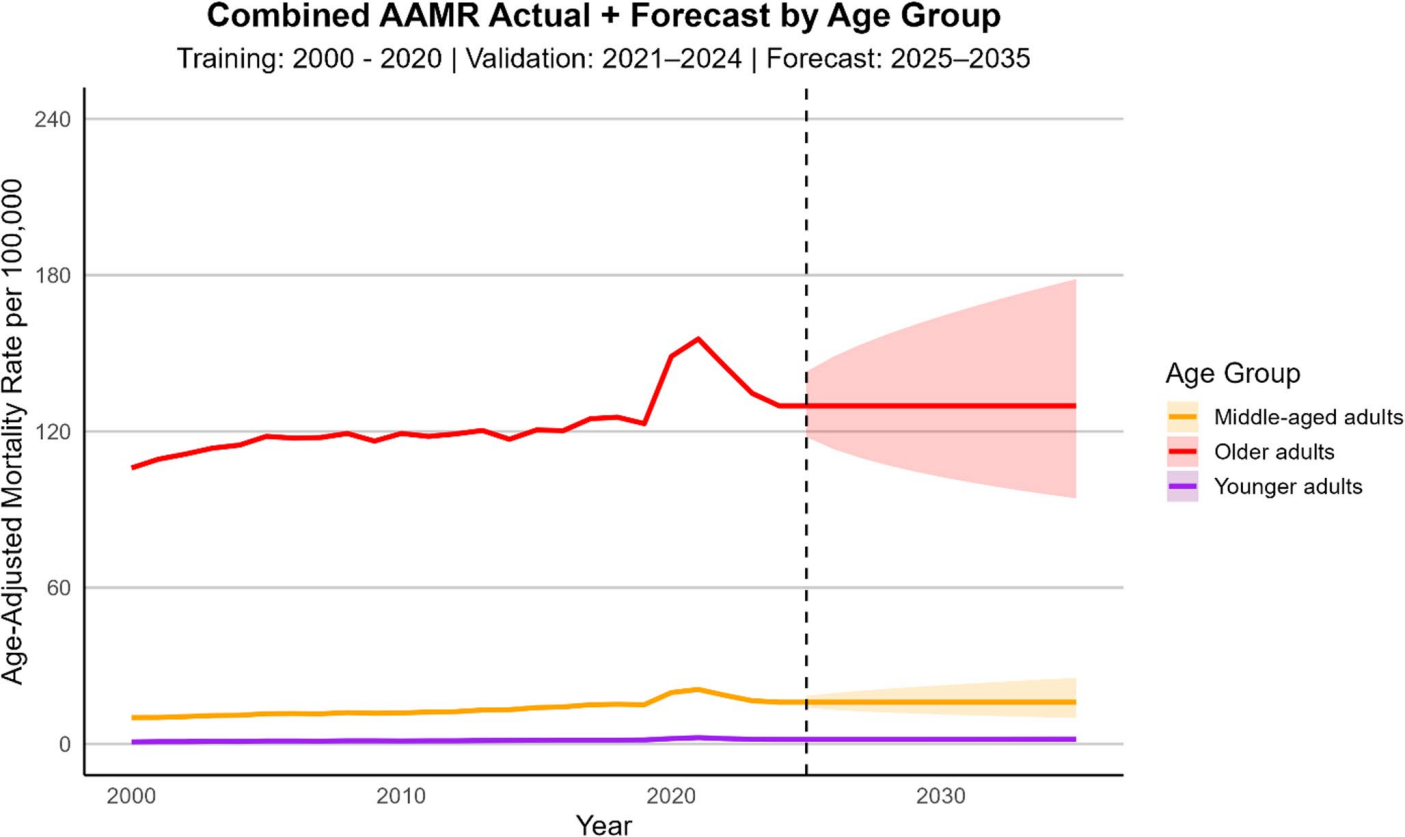
Cardiac arrest and hypertension AAMR Stratified by age per 100,000 Population

Forecasting analyses suggested plateauing age-specific mortality trends through 2035, with projected AAMRs remaining comparable to 2024 observed estimates among younger adults at 1.75 (95% CI: 0.84 to 3.10), middle-aged adults at 16.04 (95% CI: 9.94 to 25.30), and older adults at 129.79 (95% CI: 94.40 to 178.00) **(**Fig. 2**) **(Supplemental Table 9).

### CA with HTN-Related AAMR Stratified by Geographical Region

#### Stratified by Census Region

From 2000 to 2024, the AAMR increased across all U.S. census regions. In the Northeast, the AAMR rose from 31.5 in 2000 to 34.32 in 2024; in the Midwest, from 13.56 to 20.22; in the South, from 21.53 to 27.18; and in the West, from 35.8 to 47.28.

Throughout the entire study period from 2000 to 2024, the highest mean AAMR was observed in the West (mean AAMR: 43.75; 95% CI: 43.16 to 44.35), followed by the Northeast (mean AAMR: 35.35; 95% CI: 34.8 to 35.91), the South (mean AAMR: 25.05; 95% CI: 24.7 to 25.39), and the Midwest (mean AAMR: 16.62; 95% CI: 16.27 to 16.98).

**Fig. 3:**
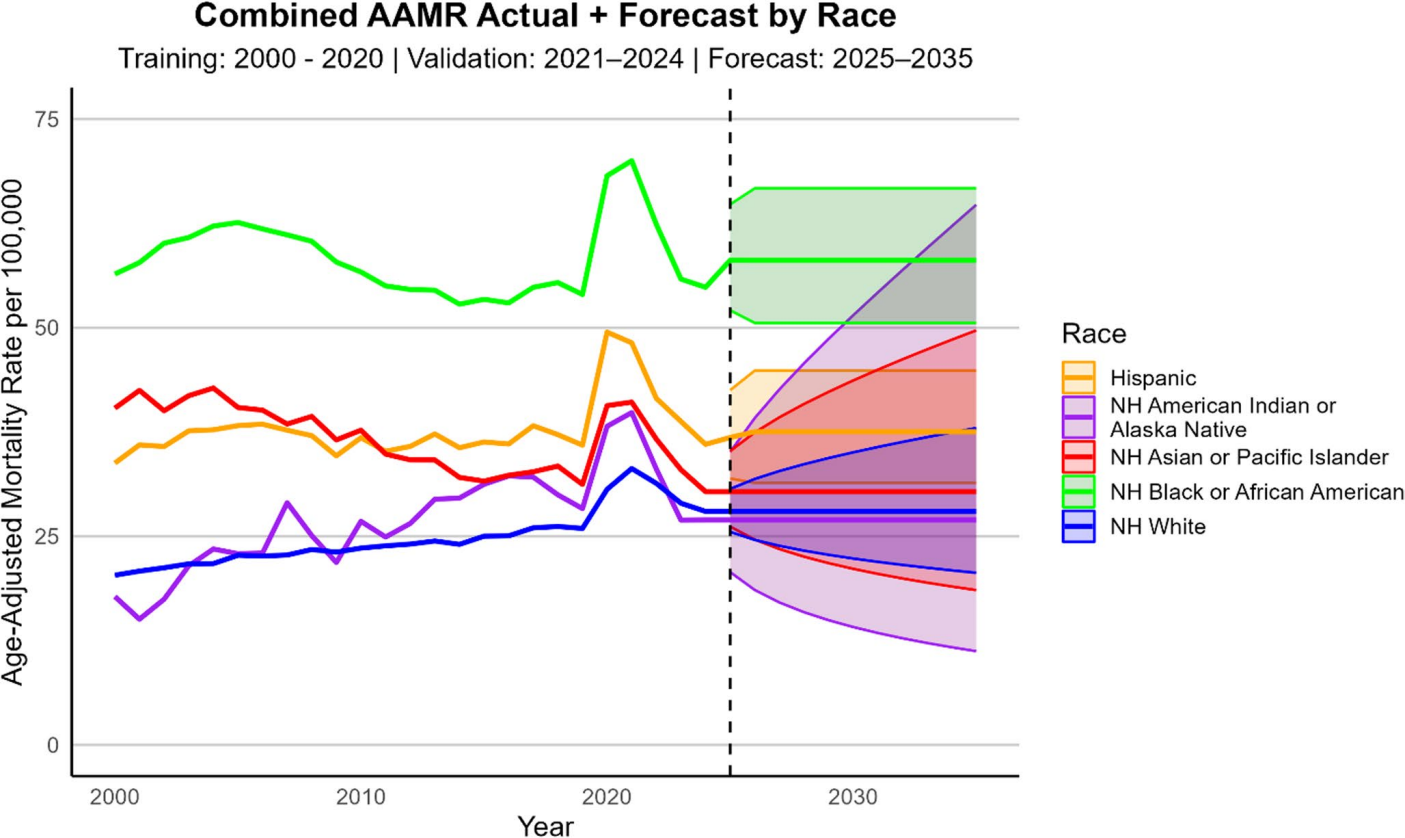
Cardiac arrest and hypertension AAMR Stratified by race 100,000 population

From 2000 to 2024, the AAMR trend remained stable with a slight rise in Northeast, South, and West, while it had a significantly greater rise in Midwest [Northeast: AAPC: 0.24; 95% CI: −0.16 to 0.69; p value = 0.22; South: AAPC: 0.73; 95% CI: 0.35 to 1.02; p value = 0.0008; West: AAPC: 0.97; 95% CI: 0.71 to 1.17; p value < 0.0001; Midwest: AAPC: 1.54; 95% CI: 1.24 to 1.79; p value < 0.0001]. (Supplemental Table 6) Fig. 3.

**Fig. 4:**
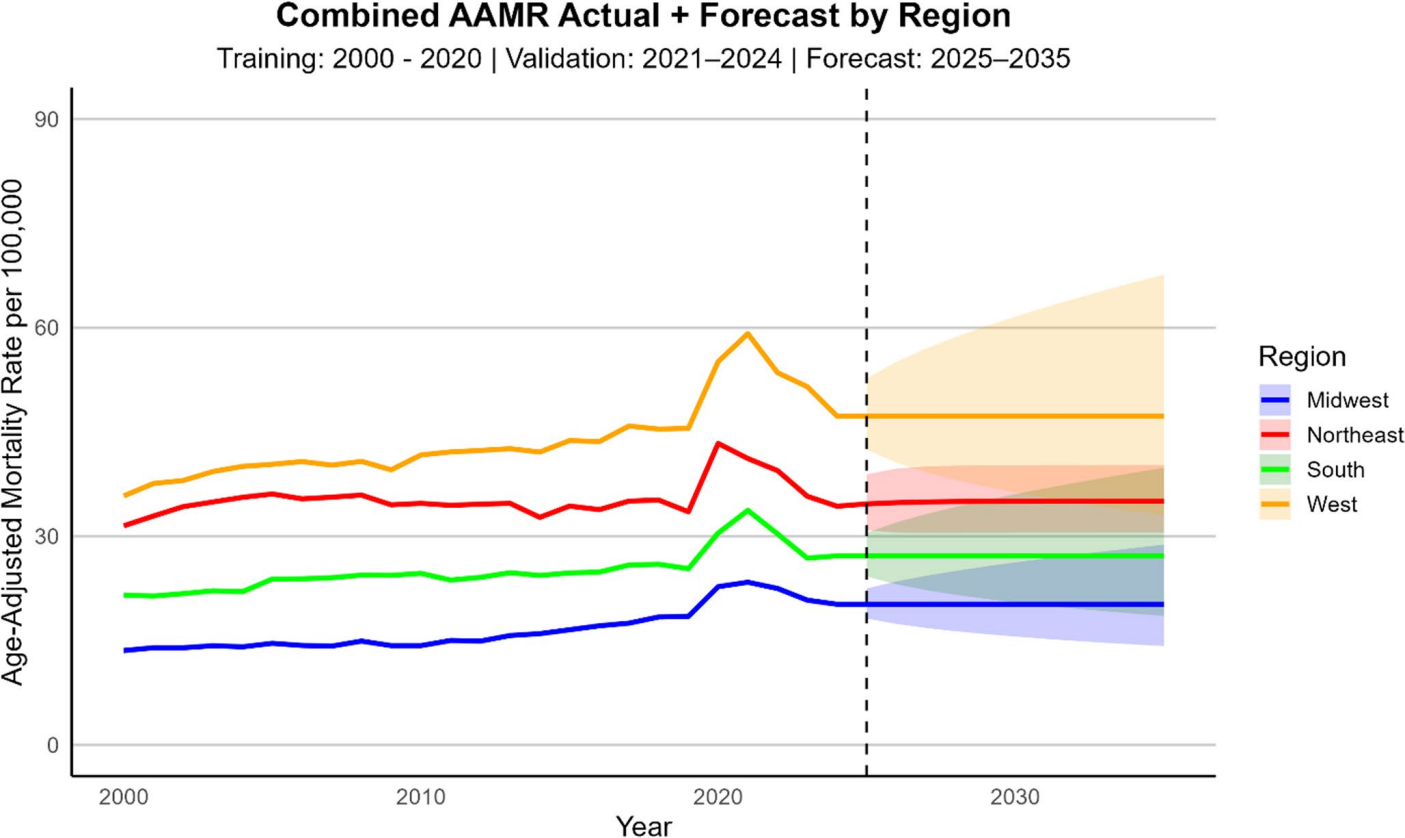
Cardiac arrest and hypertension AAMR Stratified by Census per 100,000 population

Forecasting analyses indicated relative stabilization in regional mortality trends through 2035. Projected AAMRs remained near 2024 observed levels in the Northeast at 35.05 (95% CI: 30.87 to 40.90), Midwest at 20.22 (95% CI: 14.87 to 28.90), South at 27.18 (95% CI: 18.87 to 39.90), and West at 47.28 (95% CI: 33.87 to 67.90) **(**Fig. 4**). **(Supplemental Table 9).

### Stratified by Urbanization

From 2000 to 2020, similar trends were observed across urban and rural areas. In urban areas, the AAMR increased from 25.73 in 2000 to 36.9 in 2020, similarly in rural areas it increased from 19.61 to 36.65.

Urban areas, however, showed higher AAMRs throughout the study timeline, with a mean AAMR of 28.88 for urban (95% CI: 28.63 to 29.13) and 25.5 for rural (95% CI: 24.99 to 26.01).

The AAMR trend for urban areas and rural areas showed a significant increase, with a greater rise observed in rural areas. [Urban: AAPC: 1.43, (95% CI: 0.96 to 1.7) (p value < 0.0001); Rural: AAPC: 2.8, (95% CI: 2.23 to 3.14) (p value < 0.0001)]. (Supplemental Table 7).

**Fig. 5:**
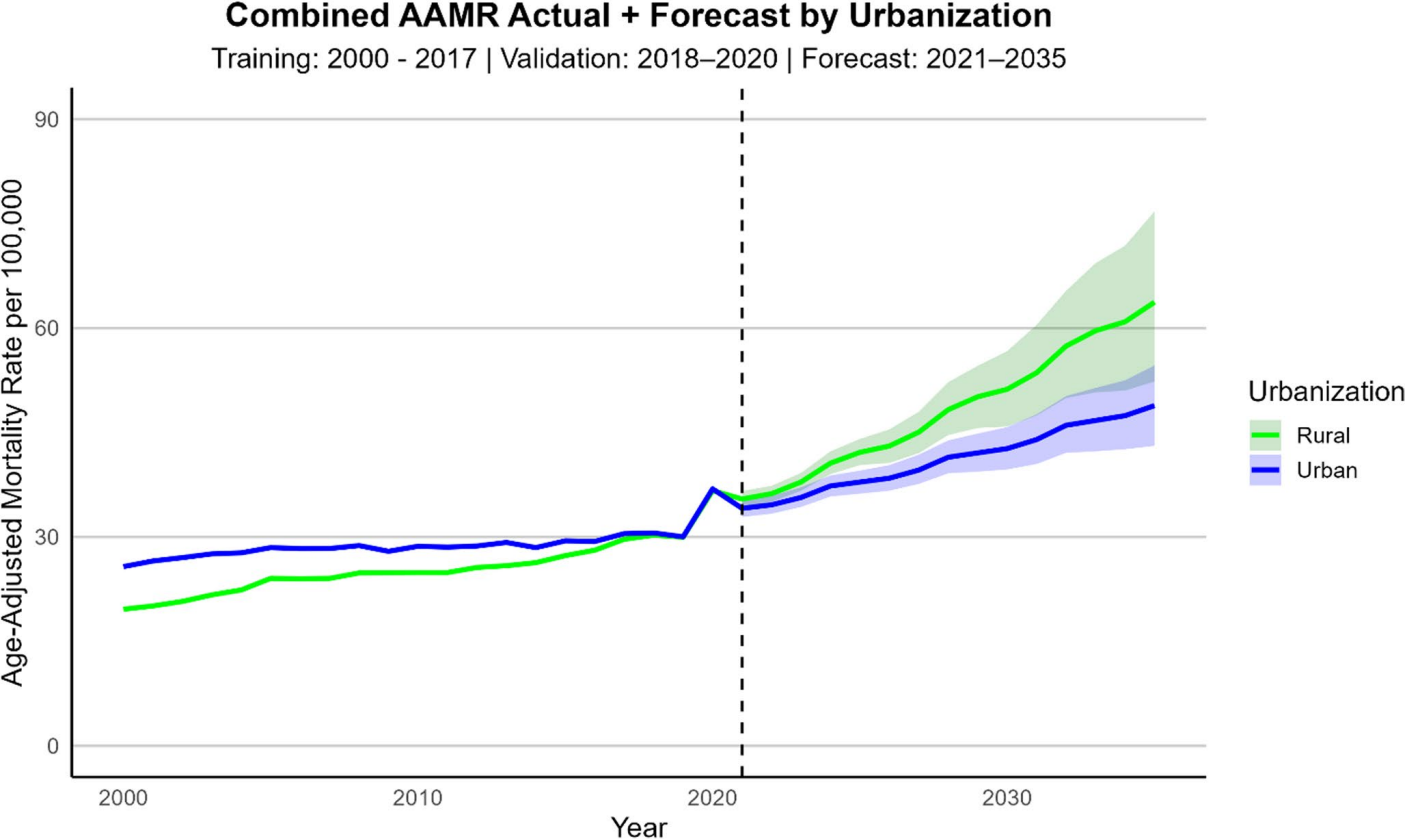
Cardiac arrest and hypertension AAMR Stratified by Urbanization per 100,000 population

Forecasting analyses projected continued increases in AAMRs through 2035, reaching 48.85 (95% CI: 43.08 to 54.30) in urban areas and 63.71 (95% CI: 52.30 to 76.24) in rural areas. (Fig. 5) (Supplemental Table 9).

### Stratified by States

**Fig. 6:**
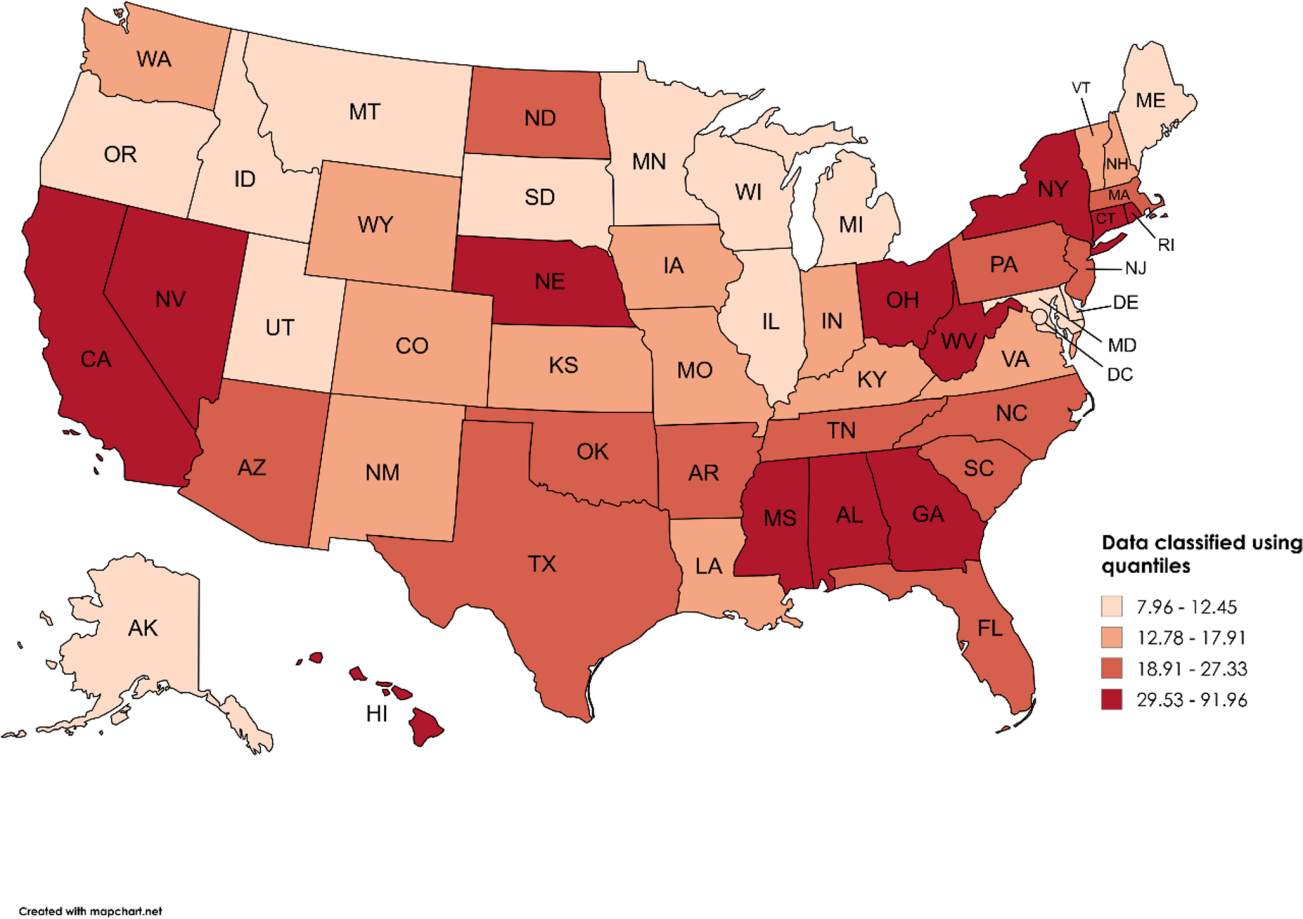
Cardiac arrest and hypertension Use-related AAMR stratified by state per 100,000 population

Disparities in AAMRs were observed among different states, with values ranging from as low as 7.96 (95% CI: 7.76 to 8.15) in Minnesota to as high as 91.96 (95% CI: 91.04 to 92.88) in Mississippi. States falling within the top 90th percentile included Mississippi, California, Alabama, Connecticut, Georgia, Hawaii, Nebraska, Nevada, New York, Ohio, Rhode Island, and West Virginia, which exhibited several-fold higher AAMRs compared to states in the lower 10th percentile which included Alaska, Delaware, Idaho, Illinois, Maine, Maryland, Michigan, Minnesota, Montana, Oregon, South Dakota, Utah, and Wisconsin (Fig. 6)(Supplemental Table 8).

## Discussion

**Fig. 7:**
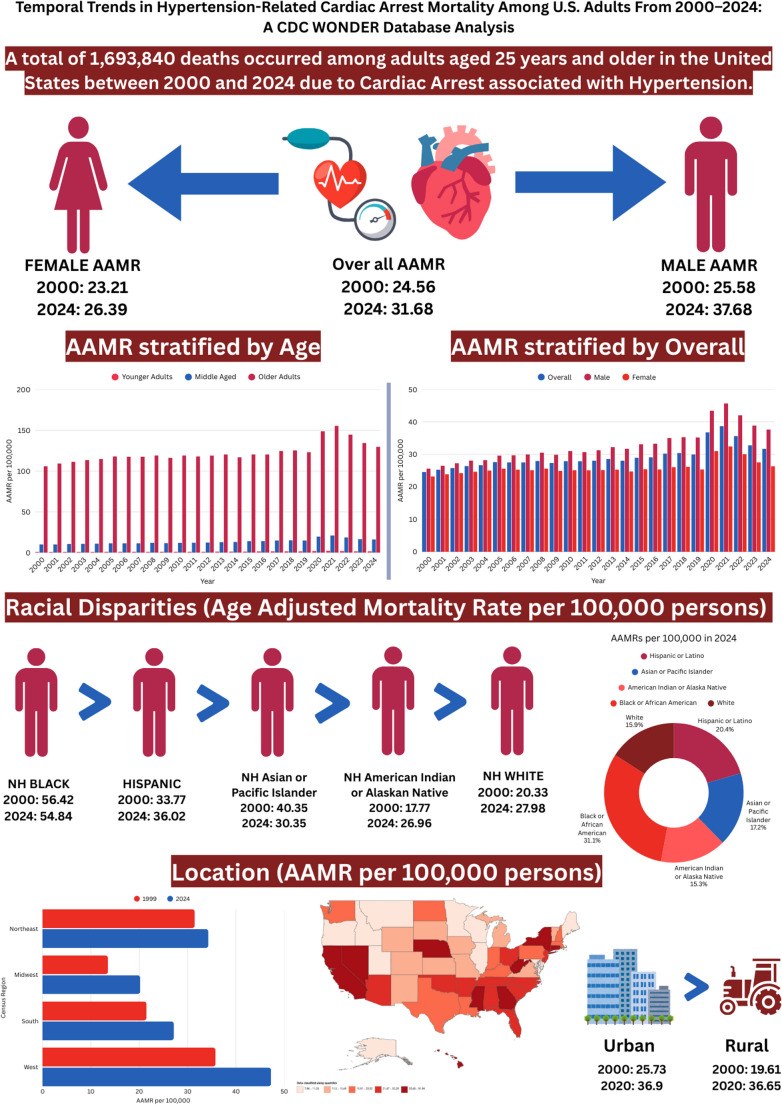
Central illustration: Cardiac arrest and hypertension among US adult

In this study, we analyzed nationwide mortality data for CA among U.S. adults (≥ 25 years) with HTN from 2000 to 2024. A total of 1,693,840 deaths were recorded, with most occurring in medical facilities, followed by homes and nursing facilities. Significant disparities were observed across sex, race, age, and geography, including Michigan, Minnesota, Montana, Oregon, South Dakota, Utah, and Wisconsin. Mortality rates were higher in men than women. By race, the highest rates were observed among non-Hispanic Black individuals, followed by Hispanics, non-Hispanic Asians/Pacific Islanders, and non-Hispanic American Indians/Alaska Natives. Older adults accounted for the highest burden. Geographically, the Western U.S. showed the highest incidence, followed by the Northeast, South, and Midwest. Urban areas had higher mortality than rural areas, although rural regions showed faster increases. At the state level, the greatest burden was observed in Mississippi, California, Alabama, Connecticut, Georgia, Hawaii, Nebraska, Nevada, New York, Ohio, Rhode Island, and West Virginia. Projections through 2035 suggest continued increases in AAMRs, particularly among older Hispanic individuals, American Indian/Alaska Native populations, and non-Hispanic Asian/Pacific Islanders. The Western and Northeastern regions are expected to experience the highest increases, while rural areas may show steeper growth compared with urban regions (Fig. 7).

HTN was associated with CA mortality in this study. Mechanistically, chronic hypertension leads to left ventricular hypertrophy and myocardial fibrosis, resulting in structural and electrical remodeling that may increase susceptibility to arrhythmias [18, 19]. These changes can impair coronary perfusion and increase myocardial oxygen demand, particularly in patients with coexisting coronary artery disease [20, 21]. Myocardial ischemia may trigger malignant ventricular arrhythmias that can culminate in CA [22, 23]. However, these mechanisms should be interpreted as biologically plausible pathways rather than causal effects due to the ecological study design. HTN has been associated with increased risk of sudden cardiac death in previous studies, including a dose-response relationship with blood pressure levels [24, 25].

The presence of comorbid conditions such as diabetes mellitus, obesity, and chronic kidney disease further increases risk through shared mechanisms including atherosclerosis, autonomic dysfunction, and electrolyte imbalance [26–28]. Structural inequities and limited access to preventive care also contribute to mortality disparities [29, 30]. A meta-analysis by Pan et al. confirmed a two-fold increased risk of CA with HTN, particularly in older adults [24].

Sex differences showed higher mortality in men, likely due to earlier coronary disease onset and higher prevalence of behavioral risk factors [31, 32]. Increasing rates among women may reflect rising cardiometabolic risk factors [33]. Racial disparities were most pronounced in non-Hispanic Black populations, reflecting higher HTN burden and structural inequities in healthcare access [34, 35]. Trends in other groups likely reflect varying cardiometabolic risk profiles and HTN control [33, 36].

Older adults had the highest burden due to cumulative cardiovascular damage and comorbidities [37, 38], while rising trends in younger populations likely reflect earlier onset of obesity, diabetes, and HTN [33]. Geographic variation may be explained by differences in risk factor prevalence, healthcare access, and socioeconomic conditions [33–41]. Higher burden states included Mississippi, Alabama, and West Virginia, while better outcomes were observed in Minnesota, Utah, and Wisconsin [42, 43]. Rural areas showed faster increases over time, indicating widening disparities in care access [44]. The rise in mortality from 2018 to 2021 likely reflects the indirect cardiovascular impact of the COVID-19 pandemic, including delayed care and increased out-of-hospital cardiac arrest incidence [45–48]. Reducing HTN-associated CA mortality requires improved hypertension detection, targeted prevention strategies, and strengthened emergency response systems, including community screening and expanded AED/CPR access [39, 43, 49].

### Limitations

This study has several limitations that should be acknowledged. Mortality estimates derived from death certificate data may be subject to underreporting and misclassification, particularly when determining contributing causes of death, and ICD-10 coding may not fully capture clinical complexity, leading to potential misclassification bias. Additionally, the CDC WONDER Multiple Cause of Death database lacks individual-level variables such as socioeconomic status, comorbidities, medication use, and access to healthcare, limiting adjustment for residual confounding. Because this is an ecological study, associations observed at the population level cannot be interpreted as individual-level causal relationships (ecological fallacy). The time-series forecasting models used (ARIMA and Prophet) also have inherent limitations, as ARIMA assumes linearity and stationarity while Prophet relies on additive decomposition and changepoint detection, which may oversimplify complex temporal patterns; therefore, future projections should be interpreted as model-based estimates rather than precise predictions. The validation period of only four years may introduce temporal bias and limit long-term trend assessment. Finally, although the dataset is nationally representative, the analysis does not account for variations in healthcare systems, emergency response capacity, or public health interventions, which may influence outcomes. Overall, findings are descriptive and hypothesis-generating and should be interpreted as associations rather than causal relationships or definitive forecasts.

## Conclusion

Hypertension-associated cardiac arrest appears to represent an important and potentially increasing public health concern in the United States, with reported disparities across sex, race, age, and geographic regions. Chronic hypertension may contribute to structural cardiac remodeling and electrical instability, which, together with cardiometabolic dysfunction, could increase susceptibility to adverse cardiac outcomes. The observed differences across populations may reflect a combination of biological vulnerability and structural inequities in healthcare access and delivery. While some subgroups show improving trends, increasing mortality among younger adults and rural populations remains notable and may suggest evolving epidemiological patterns. The rise in mortality during the COVID-19 pandemic may also reflect vulnerability of hypertensive populations to disruptions in healthcare systems. Addressing this burden may require earlier detection of hypertension, improved risk factor management, more equitable access to care, strengthened emergency response systems, and targeted population-based interventions.

## Supplementary Information

Below is the link to the electronic supplementary material.

Supplementary File 1 (DOCX 104 KB)

## Data Availability

The data supporting the findings of this study were obtained from the CDC WONDER online database (Centers for Disease Control and Prevention Wide-ranging Online Data for Epidemiologic Research). The datasets used and analyzed during the current study are publicly available and can be accessed at [CDC WONDER] ([https://wonder.cdc.gov](http:/wonder.cdc.gov)).

## Scientific category

Original Research.
