# Supplementary material for: Temporal Trends and Forecasted Cardiac Arrest and Hypertension-Related Mortality in U.S. Adults, 2000–2035: A Nationwide CDC WONDER Multiple Cause-of-Death Study
Source: J Epidemiol Glob Health. 2026 May 25;16(1):94. doi: 10.1007/s44197-026-00588-x (PMC13391468; doi:10.1007/s44197-026-00588-x)
Supplement: Supplementary file 1 — (DOCX 104 KB) [file 44197_2026_588_MOESM1_ESM.docx]

**Supplemental Table 1, Deaths Among U.S. Adults with Cardiac Arrest and Hypertension Recorded on the Death Certificate, Stratified by Sex and Race, 2000 to 2024**

| **Supplemental Table 1, Deaths Among U.S. Adults with Cardiac Arrest and Hypertension Recorded on the Death Certificate, Stratified by Sex and Race, 2000 to 2024** | | | | | | | | | |
| --- | --- | --- | --- | --- | --- | --- | --- | --- | --- |
| **Deaths** | | | | | | | | | |
| **Year** | **Overall** | **Women** | **Men** | **NH White** | **NH Black** | **NH American Indian or Alaska Native** | **NH Asian or Pacific Islander** | **Hispanic** | **Population** |
| **2000** | 43800 | 25710 | 18090 | 30656 | 8412 | 132 | 1573 | 2876 | 181984640 |
| **2001** | 45876 | 26753 | 19123 | 31800 | 8784 | 113 | 1799 | 3207 | 184305128 |
| **2002** | 47552 | 27511 | 20041 | 32767 | 9275 | 141 | 1788 | 3390 | 186208028 |
| **2003** | 49596 | 28399 | 21197 | 33973 | 9569 | 175 | 1970 | 3736 | 188090429 |
| **2004** | 50804 | 29149 | 21655 | 34420 | 9983 | 200 | 2107 | 3940 | 190205384 |
| **2005** | 53673 | 30295 | 23378 | 36606 | 10341 | 199 | 2163 | 4257 | 192551384 |
| **2006** | 54641 | 30568 | 24073 | 37102 | 10500 | 213 | 2261 | 4486 | 195019359 |
| **2007** | 55812 | 30961 | 24851 | 37952 | 10614 | 255 | 2315 | 4615 | 197403777 |
| **2008** | 58076 | 32052 | 26024 | 39644 | 10812 | 238 | 2477 | 4813 | 199795090 |
| **2009** | 57904 | 31736 | 26168 | 39761 | 10637 | 224 | 2454 | 4728 | 202107016 |
| **2010** | 60175 | 32595 | 27580 | 41144 | 10676 | 281 | 2654 | 5219 | 203891983 |
| **2011** | 61784 | 33382 | 28402 | 42509 | 10727 | 286 | 2666 | 5404 | 206592936 |
| **2012** | 63799 | 34060 | 29739 | 43635 | 10991 | 315 | 2813 | 5823 | 208826037 |
| **2013** | 66561 | 35009 | 31552 | 45099 | 11419 | 362 | 3019 | 6443 | 211085314 |
| **2014** | 66753 | 34688 | 32065 | 45038 | 11460 | 390 | 3067 | 6555 | 213809280 |
| **2015** | 70705 | 36354 | 34351 | 47530 | 12020 | 424 | 3237 | 7167 | 216553817 |
| **2016** | 72300 | 36934 | 35366 | 48249 | 12341 | 453 | 3495 | 7466 | 218641417 |
| **2017** | 76993 | 38709 | 38284 | 50839 | 13182 | 483 | 3789 | 8371 | 221447331 |
| **2018** | 79130 | 39554 | 39576 | 52073 | 13703 | 471 | 4065 | 8488 | 223311190 |
| **2019** | 79405 | 39001 | 40404 | 52390 | 13753 | 457 | 4014 | 8546 | 224981167 |
| **2020** | 99136 | 48099 | 51037 | 62241 | 17899 | 656 | 5511 | 12442 | 226635013 |
| **2021** | 101319 | 48140 | 53179 | 64066 | 18055 | 629 | 5413 | 12324 | 228238412 |
| **2022** | 97734 | 47305 | 50429 | 63629 | 16535 | 551 | 5216 | 10969 | 229508599 |
| **2023** | 90443 | 42990 | 47453 | 58606 | 15053 | 461 | 4831 | 10598 | 231529762 |
| **2024** | 89869 | 42408 | 47461 | 57778 | 15376 | 487 | 4812 | 10493 | 235615087 |
| **Total** | **1693840** | **882362** | **811478** | **1129507** | **302117** | **8596** | **79509** | **166356** | **5218337580** |
| NH = Non-Hispanic | | | | | | | | | |

**Supplemental Table 2, Deaths Among U.S. Adults with Cardiac Arrest and Hypertension Recorded on the Death Certificate, Stratified by Place of Death, 2000 to 2024**

| **Supplemental Table 2, Deaths Among U.S. Adults with Cardiac Arrest and Hypertension Recorded on the Death Certificate, Stratified by Place of Death, 2000 to 2024** | | | | |
| --- | --- | --- | --- | --- |
| **Deaths** | | | | |
| **Year** | **Medical Facility** | **Nursing Home/Long-term Care Facility** | **Decedents Home** | **Hospice Facility** |
| **2000** | 23702 | 9744 | 9301 | - |
| **2001** | 24373 | 10541 | 9772 | - |
| **2002** | 24973 | 10954 | 10298 | - |
| **2003** | 25621 | 11477 | 11062 | 45 |
| **2004** | 25650 | 11829 | 11583 | 99 |
| **2005** | 26824 | 12839 | 12288 | 251 |
| **2006** | 27082 | 12599 | 13191 | 283 |
| **2007** | 27142 | 12917 | 13645 | 349 |
| **2008** | 27317 | 12719 | 14212 | 483 |
| **2009** | 25894 | 12695 | 14556 | 616 |
| **2010** | 27722 | 13478 | 16378 | 754 |
| **2011** | 27889 | 14215 | 16928 | 821 |
| **2012** | 27978 | 14545 | 18275 | 892 |
| **2013** | 28304 | 14976 | 19664 | 1131 |
| **2014** | 28249 | 14691 | 20320 | 856 |
| **2015** | 29355 | 15453 | 22215 | 860 |
| **2016** | 30097 | 15169 | 23009 | 842 |
| **2017** | 31414 | 16362 | 24758 | 1039 |
| **2018** | 31664 | 16310 | 26502 | 1212 |
| **2019** | 31610 | 15341 | 27285 | 1398 |
| **2020** | 38628 | 18248 | 36213 | 1275 |
| **2021** |  |  |  |  |
| **2022** |  |  |  |  |
| **2023** |  |  |  |  |
| **2024** |  |  |  |  |
| **Total** | 603544 | 292049 | 377146 | 13206 |

**Supplemental Table 3. Overall and Sex-Stratified Age-Adjusted Mortality Rates per 100,000 Among U.S. Adults with Cardiac Arrest and Hypertension Listed on the Death Certificate, 2000–2024.**

| **Supplemental Table 3. Overall and Sex-Stratified Age-Adjusted Mortality Rates per 100,000 Among U.S. Adults with Cardiac Arrest and Hypertension Listed on the Death Certificate, 2000–2024.** | | | |
| --- | --- | --- | --- |
| **Age-Adjusted Rate (95% CI)** | | | |
| **Year** | **Men** | **Women** | **Overall** |
| **2000** | 25.58 (25.20–25.96) | 23.21 (22.93–23.50) | 24.56 (24.33–24.79) |
| **2001** | 26.5 (26.120–26.88) | 23.86 (23.57–24.150) | 25.28 (25.05–25.51) |
| **2002** | 27.27 (26.880–27.65) | 24.23 (23.94–24.520) | 25.78 (25.55–26.02) |
| **2003** | 28.1 (27.720–28.49) | 24.66 (24.37–24.950) | 26.43 (26.2–26.67) |
| **2004** | 28.19 (27.810–28.57) | 25.01 (24.72–25.30) | 26.68 (26.45–26.91) |
| **2005** | 29.6 (29.210–29.98) | 25.58 (25.29–25.870) | 27.62 (27.38–27.85) |
| **2006** | 29.75 (29.370–30.13) | 25.32 (25.03–25.610) | 27.51 (27.27–27.74) |
| **2007** | 29.98 (29.60–30.36) | 25.13 (24.85–25.420) | 27.49 (27.26–27.72) |
| **2008** | 30.55 (30.170–30.93) | 25.61 (25.33–25.890) | 28 (27.77–28.23) |
| **2009** | 29.92 (29.550–30.29) | 24.87 (24.59–25.150) | 27.34 (27.12–27.57) |
| **2010** | 31.05 (30.680–31.42) | 25.17 (24.89–25.450) | 27.93 (27.7–28.15) |
| **2011** | 30.74 (30.380–31.11) | 25.12 (24.84–25.390) | 27.86 (27.64–28.08) |
| **2012** | 31.3 (30.940–31.67) | 25.19 (24.92–25.460) | 28.09 (27.87–28.31) |
| **2013** | 32.23 (31.870–32.6) | 25.33 (25.06–25.60) | 28.61 (28.39–28.83) |
| **2014** | 31.75 (31.390–32.1) | 24.7 (24.43–24.960) | 28.02 (27.81–28.24) |
| **2015** | 33.12 (32.760–33.48) | 25.39 (25.12–25.650) | 29.01 (28.8–29.23) |
| **2016** | 33.25 (32.90–33.6) | 25.36 (25.1–25.620) | 29.08 (28.87–29.3) |
| **2017** | 35.06 (34.70–35.42) | 26.05 (25.78–26.310) | 30.26 (30.05–30.48) |
| **2018** | 35.31 (34.950–35.66) | 26.18 (25.92–26.450) | 30.44 (30.23–30.65) |
| **2019** | 35.21 (34.860–35.56) | 25.37 (25.11–25.620) | 29.93 (29.72–30.14) |
| **2020** | 43.46 (43.070–43.84) | 30.98 (30.7–31.270) | 36.81 (36.57–37.04) |
| **2021** | 45.71 (45.310–46.11) | 32.49 (32.19–32.780) | 38.7 (38.46–38.94) |
| **2022** | 42.08 (41.710–42.46) | 30.09 (29.82–30.370) | 35.67 (35.44–35.89) |
| **2023** | 38.87 (38.510–39.23) | 27.56 (27.29–27.820) | 32.83 (32.62–33.05) |
| **2024** | 37.68 (37.340–38.03) | 26.39 (26.14–26.650) | 31.68 (31.47–31.89) |
| **Mean** | 32.89 (32.52–33.26) | 25.95 (25.68–26.23) | 29.26 (29.04–29.49) |

**Supplemental Table 4. Age-Stratified Age-Adjusted Mortality Rates per 100,000 Among U.S. Adults with Cardiac Arrest and Hypertension Listed on the Death Certificate, 2000–2024.**

| Supplemental Table 4. Age-Stratified Age-Adjusted Mortality Rates per 100,000 Among U.S. Adults with Cardiac Arrest and Hypertension Listed on the Death Certificate, 2000–2024. | | | |
| --- | --- | --- | --- |
| Age-Adjusted Rate (95% CI) | | | |
| Year | **Younger Adults (25-44)** | **Middle-aged Adults (45-64)** | **Older Adults (65+)** |
| 2000 | 0.80 (0.74 - 0.86) | 10.14 (9.89 - 10.39) | 105.98 (104.90 - 107.06) |
| 2001 | 0.90 (0.84 - 0.96) | 10.18 (9.93 - 10.42) | 109.35 (108.260 - 110.44) |
| 2002 | 0.90 (0.84 - 0.96) | 10.51 (10.27 - 10.76) | 111.32 (110.230 - 112.42) |
| 2003 | 1.050 (0.98 - 1.13) | 10.91 (10.67 - 11.16) | 113.59 (112.490 - 114.69) |
| 2004 | 1.010 (0.94 - 1.08) | 11.01 (10.77 - 11.26) | 114.76 (113.670 - 115.86) |
| 2005 | 1.160 (1.09 - 1.24) | 11.63 (11.39 - 11.88) | 118.12 (117.020 - 119.23) |
| 2006 | 1.160 (1.09 - 1.24) | 11.69 (11.45 - 11.93) | 117.44 (116.350 - 118.54) |
| 2007 | 1.110 (1.04 - 1.18) | 11.59 (11.36 - 11.83) | 117.66 (116.580 - 118.74) |
| 2008 | 1.220 (1.14 - 1.3) | 12.05 (11.81 - 12.29) | 119.22 (118.150 - 120.3) |
| 2009 | 1.220 (1.14 - 1.3) | 11.79 (11.56 - 12.03) | 116.3 (115.240 - 117.35) |
| 2010 | 1.160 (1.09 - 1.24) | 11.91 (11.68 - 12.15) | 119.22 (118.160 - 120.28) |
| 2011 | 1.220 (1.14 - 1.3) | 12.27 (12.04 - 12.5) | 118.1 (117.060 - 119.14) |
| 2012 | 1.220 (1.14 - 1.3) | 12.41 (12.17 - 12.64) | 119.04 (118.010 - 120.07) |
| 2013 | 1.320 (1.24 - 1.4) | 13.04 (12.8 - 13.28) | 120.37 (119.350 - 121.4) |
| 2014 | 1.380 (1.3 - 1.46) | 13.16 (12.92 - 13.4) | 117 (1160 - 118) |
| 2015 | 1.440 (1.35 - 1.52) | 13.96 (13.71 - 14.2) | 120.54 (119.540 - 121.54) |
| 2016 | 1.480 (1.4 - 1.57) | 14.24 (13.99 - 14.48) | 120.3 (119.320 - 121.29) |
| 2017 | 1.480 (1.4 - 1.57) | 15.07 (14.82 - 15.33) | 124.87 (123.870 - 125.86) |
| 2018 | 1.480 (1.4 - 1.57) | 15.25 (14.99 - 15.51) | 125.46 (124.480 - 126.44) |
| 2019 | 1.550 (1.46 - 1.63) | 15.09 (14.83 - 15.34) | 122.99 (122.030 - 123.95) |
| 2020 | 2.060 (1.96 - 2.16) | 19.74 (19.45 - 20.03) | 148.77 (147.730 - 149.81) |
| 2021 | 2.430 (2.32 - 2.54) | 20.93 (20.63 - 21.23) | 155.49 (154.40 - 156.58) |
| 2022 | 2.060 (1.97 - 2.16) | 18.66 (18.38 - 18.95) | 144.83 (143.810 - 145.85) |
| 2023 | 1.80 (1.71 - 1.89) | 16.59 (16.32 - 16.86) | 134.6 (133.620 - 135.58) |
| 2024 | 1.750 (1.66 - 1.83) | 16.04 (15.77 - 16.3) | 129.79 (128.850 - 130.74) |
| Mean | **1.37 (1.30 - 1.46)** | **13.59 (13.34 - 13.85)** | **122.60 (121.56 - 123.65)** |

**Supplemental Table 5. Race-Stratified Age-Adjusted Mortality Rates per 100,000 Among U.S. Adults with Cardiac Arrest and Hypertension Listed on the Death Certificate, 2000–2024.**

| **Supplemental Table 5. Race-Stratified Age-Adjusted Mortality Rates per 100,000 Among U.S. Adults with Cardiac Arrest and Hypertension Listed on the Death Certificate, 2000–2024.** | | | | | |
| --- | --- | --- | --- | --- | --- |
| **Age-Adjusted Rate (95% CI)** | | | | | |
| **Year** | **NH White** | **NH Black** | **Hispanic** | **NH American Indian or Alaska Native** | **NH Asian or Pacific Islanders** |
| **2000** | 20.33 (20.10–20.56) | 56.42 (55.20–57.64) | 33.77 (32.48–35.05) | 17.77 (14.60–20.94) | 40.35 (38.28–42.42) |
| **2001** | 20.82 (20.60–21.05) | 57.8 (56.570–59.02) | 35.95 (34.65–37.24) | 15.06 (12.120–18) | 42.48 (40.45–44.52) |
| **2002** | 21.22 (20.990–21.45) | 60.12 (58.880–61.36) | 35.76 (34.5–37.01) | 17.45 (14.410–20.49) | 40.04 (38.12–41.96) |
| **2003** | 21.69 (21.450–21.92) | 60.8 (59.560–62.04) | 37.63 (36.37–38.89) | 21.45 (18.060–24.85) | 41.84 (39.93–43.75) |
| **2004** | 21.72 (21.490–21.95) | 62.17 (60.920–63.41) | 37.76 (36.53–38.99) | 23.47 (19.980–26.96) | 42.76 (40.88–44.64) |
| **2005** | 22.72 (22.490–22.96) | 62.6 (61.370–63.83) | 38.26 (37.07–39.46) | 22.91 (19.520–26.3) | 40.43 (38.68–42.19) |
| **2006** | 22.62 (22.390–22.85) | 61.83 (60.620–63.04) | 38.43 (37.26–39.6) | 22.99 (19.680–26.3) | 40.11 (38.41–41.81) |
| **2007** | 22.75 (22.520–22.98) | 61.12 (59.930–62.31) | 37.71 (36.58–38.84) | 29.02 (25.240–32.8) | 38.45 (36.85–40.06) |
| **2008** | 23.41 (23.180–23.64) | 60.35 (59.190–61.52) | 37.06 (35.97–38.15) | 25.08 (21.680–28.49) | 39.36 (37.77–40.94) |
| **2009** | 23.1 (22.870–23.33) | 57.86 (56.730–58.99) | 34.64 (33.62–35.66) | 21.86 (18.80–24.93) | 36.55 (35.07–38.02) |
| **2010** | 23.58 (23.350–23.8) | 56.67 (55.570–57.77) | 36.81 (35.77–37.84) | 26.79 (23.440–30.14) | 37.71 (36.25–39.18) |
| **2011** | 23.85 (23.620–24.07) | 55 (53.930–56.07) | 35.19 (34.22–36.16) | 24.91 (21.830–28) | 34.85 (33.5–36.2) |
| **2012** | 24.06 (23.830–24.28) | 54.57 (53.520–55.62) | 35.77 (34.83–36.72) | 26.53 (23.420–29.64) | 34.16 (32.87–35.44) |
| **2013** | 24.43 (24.20–24.66) | 54.49 (53.460–55.52) | 37.26 (36.33–38.2) | 29.44 (26.240–32.63) | 34.14 (32.9–35.37) |
| **2014** | 24.03 (23.80–24.25) | 52.81 (51.810–53.8) | 35.61 (34.72–36.49) | 29.58 (26.490–32.67) | 32.05 (30.89–33.2) |
| **2015** | 25.01 (24.780–25.24) | 53.39 (52.410–54.38) | 36.3 (35.43–37.16) | 31.23 (28.120–34.34) | 31.61 (30.51–32.71) |
| **2016** | 25.05 (24.820–25.28) | 52.96 (520–53.92) | 36.06 (35.22–36.91) | 32.23 (29.140–35.32) | 32.31 (31.23–33.4) |
| **2017** | 26.01 (25.780–26.24) | 54.83 (53.870–55.8) | 38.24 (37.4–39.08) | 32.09 (29.110–35.07) | 32.73 (31.68–33.79) |
| **2018** | 26.17 (25.940–26.4) | 55.4 (54.450–56.35) | 37.18 (36.37–38) | 29.97 (27.170–32.77) | 33.41 (32.37–34.45) |
| **2019** | 25.94 (25.720–26.17) | 53.98 (53.060–54.91) | 35.92 (35.14–36.7) | 28.32 (25.640–30.99) | 31.22 (30.24–32.2) |
| **2020** | 30.63 (30.390–30.88) | 68.2 (67.180–69.23) | 49.46 (48.57–50.35) | 38.18 (35.170–41.19) | 40.65 (39.57–41.74) |
| **2021** | 33.12 (32.860–33.39) | 70 (68.950–71.06) | 48.18 (47.3–49.07) | 39.82 (36.670–43.18) | 41.07 (39.98–42.2) |
| **2022** | 31.34 (31.10–31.59) | 62.38 (61.410–63.37) | 41.52 (40.72–42.33) | 33.06 (30.30–36.03) | 36.64 (35.65–37.66) |
| **2023** | 28.96 (28.720–29.2) | 55.82 (54.90–56.74) | 38.76 (38–39.53) | 26.94 (24.480–29.59) | 32.98 (32.05–33.93) |
| **2024** | 27.98 (27.750–28.21) | 54.84 (53.950–55.74) | 36.02 (35.32–36.74) | 26.96 (24.570–29.53) | 30.35 (29.49–31.22) |
| **Mean** | 24.82 (24.59–25.05) | 58.26 (57.18–59.34) | 37.81 (36.81–38.81) | 26.92 (23.84–30.05) | 36.73 (35.34–38.12) |
| NH = Non-Hispanic | | | | | |

**Supplemental Table 6. Census Region–Stratified Age-Adjusted Mortality Rates per 100,000 Among U.S. Adults with Cardiac Arrest and Hypertension Listed on the Death Certificate, 2000–2024.**

| **Supplemental Table 6. Census Region–Stratified Age-Adjusted Mortality Rates per 100,000 Among U.S. Adults with Cardiac Arrest and Hypertension Listed on the Death Certificate, 2000–2024.** | | | | |
| --- | --- | --- | --- | --- |
|  | **Census Region: Northeast** | **Census Region:**  **Midwest** | **Census Region:**  **South** | **Census Region: West** |
| **Year** | **Age-Adjusted Rate (95% CI)** | **Age-Adjusted Rate (95% CI)** | **Age-Adjusted Rate (95% CI)** | **Age-Adjusted Rate (95% CI)** |
| **2000** | 31.50 (30.94–32.07) | 13.56 (13.21–13.91) | 21.53 (21.17–21.90) | 35.80 (35.18–36.43) |
| **2001** | 32.910 (32.34–33.49) | 13.96 (13.61–14.32) | 21.41 (21.05–21.770) | 37.590 (36.96–38.22) |
| **2002** | 34.260 (33.68–34.84) | 13.96 (13.61–14.31) | 21.75 (21.39–22.110) | 38.020 (37.39–38.65) |
| **2003** | 34.930 (34.34–35.51) | 14.25 (13.89–14.6) | 22.15 (21.79–22.510) | 39.310 (38.67–39.94) |
| **2004** | 35.60 (35.02–36.19) | 14.1 (13.76–14.45) | 22.03 (21.67–22.380) | 40.050 (39.42–40.69) |
| **2005** | 36.080 (35.49–36.66) | 14.6 (14.25–14.96) | 23.85 (23.48–24.210) | 40.350 (39.72–40.97) |
| **2006** | 35.380 (34.8–35.96) | 14.29 (13.95–14.64) | 23.87 (23.51–24.230) | 40.740 (40.12–41.36) |
| **2007** | 35.620 (35.04–36.19) | 14.2 (13.86–14.54) | 24.04 (23.68–24.390) | 40.230 (39.62–40.84) |
| **2008** | 35.920 (35.34–36.49) | 14.92 (14.58–15.27) | 24.41 (24.06–24.770) | 40.770 (40.16–41.37) |
| **2009** | 34.520 (33.96–35.08) | 14.27 (13.93–14.61) | 24.37 (24.02–24.720) | 39.560 (38.97–40.15) |
| **2010** | 34.730 (34.17–35.29) | 14.27 (13.94–14.61) | 24.66 (24.31–25.010) | 41.690 (41.09–42.29) |
| **2011** | 34.450 (33.89–35) | 15.01 (14.67–15.35) | 23.7 (23.36–24.040) | 42.130 (41.54–42.72) |
| **2012** | 34.620 (34.07–35.17) | 14.92 (14.58–15.26) | 24.09 (23.75–24.430) | 42.340 (41.75–42.92) |
| **2013** | 34.730 (34.18–35.28) | 15.72 (15.37–16.06) | 24.76 (24.42–25.090) | 42.590 (42.01–43.17) |
| **2014** | 32.720 (32.19–33.25) | 15.99 (15.64–16.34) | 24.36 (24.02–24.690) | 42.110 (41.55–42.68) |
| **2015** | 34.340 (33.8–34.88) | 16.55 (16.2–16.9) | 24.73 (24.4–25.060) | 43.760 (43.2–44.33) |
| **2016** | 33.830 (33.3–34.36) | 17.12 (16.77–17.47) | 24.85 (24.52–25.180) | 43.620 (43.07–44.18) |
| **2017** | 35.060 (34.52–35.59) | 17.5 (17.15–17.85) | 25.86 (25.53–26.190) | 45.850 (45.29–46.42) |
| **2018** | 35.210 (34.68–35.74) | 18.39 (18.03–18.75) | 25.97 (25.64–26.290) | 45.410 (44.85–45.97) |
| **2019** | 33.520 (33–34.03) | 18.48 (18.13–18.84) | 25.34 (25.02–25.660) | 45.520 (44.97–46.07) |
| **2020** | 43.350 (42.77–43.93) | 22.75 (22.36–23.15) | 30.46 (30.12–30.810) | 55.110 (54.52–55.71) |
| **2021** | 41.180 (40.61–41.76) | 23.39 (22.99–23.81) | 33.72 (33.36–34.090) | 59.130 (58.5–59.77) |
| **2022** | 39.440 (38.9–40) | 22.49 (22.1–22.89) | 30.33 (29.99–30.670) | 53.510 (52.93–54.1) |
| **2023** | 35.750 (35.23–36.28) | 20.81 (20.44–21.19) | 26.85 (26.53–27.170) | 51.50 (50.93–52.07) |
| **2024** | 34.320 (33.82–34.83) | 20.22 (19.86–20.6) | 27.18 (26.87–27.50) | 47.280 (46.75–47.82) |
| **Mean** | 35.36 (34.80–35.91) | 16.63 (16.28–16.99) | 25.05 (24.71–25.39) | 43.76 (43.17–44.35) |

**Supplemental Table 7. Urban–Rural Stratified Age-Adjusted Mortality Rates per 100,000 Among U.S. Adults with Cardiac Arrest and Hypertension Listed on the Death Certificate, 2000–2020.**

| **Supplemental Table 7. Urban–Rural Stratified Age-Adjusted Mortality Rates per 100,000 Among U.S. Adults with Cardiac Arrest and Hypertension Listed on the Death Certificate, 2000–2020.** | | |
| --- | --- | --- |
| **Age-Adjusted Rate (95% CI)** | | |
| **Year** | **Rural** | **Urban** |
| **2000** | 19.61 (19.14 - 20.08) | 25.73 (25.47 - 25.99) |
| **2001** | 20.08 (19.6 - 20.55) | 26.54 (26.28 - 26.8) |
| **2002** | 20.73 (20.25 - 21.22) | 27.02 (26.76 - 27.29) |
| **2003** | 21.67 (21.18 - 22.16) | 27.56 (27.3 - 27.83) |
| **2004** | 22.38 (21.88 - 22.88) | 27.71 (27.45 - 27.97) |
| **2005** | 24.04 (23.53 - 24.55) | 28.46 (28.2 - 28.73) |
| **2006** | 23.99 (23.48 - 24.5) | 28.32 (28.06 - 28.58) |
| **2007** | 24.04 (23.53 - 24.54) | 28.33 (28.07 - 28.58) |
| **2008** | 24.82 (24.31 - 25.33) | 28.75 (28.49 - 29) |
| **2009** | 24.86 (24.35 - 25.36) | 27.93 (27.68 - 28.18) |
| **2010** | 24.89 (24.39 - 25.4) | 28.65 (28.4 - 28.91) |
| **2011** | 24.88 (24.38 - 25.38) | 28.53 (28.28 - 28.78) |
| **2012** | 25.61 (25.11 - 26.12) | 28.69 (28.44 - 28.93) |
| **2013** | 25.86 (25.35 - 26.36) | 29.2 (28.96 - 29.44) |
| **2014** | 26.3 (25.79 - 26.81) | 28.46 (28.22 - 28.69) |
| **2015** | 27.31 (26.79 - 27.83) | 29.43 (29.19 - 29.67) |
| **2016** | 28.09 (27.57 - 28.61) | 29.34 (29.11 - 29.58) |
| **2017** | 29.66 (29.12 - 30.19) | 30.47 (30.23 - 30.71) |
| **2018** | 30.25 (29.71 - 30.78) | 30.57 (30.34 - 30.81) |
| **2019** | 29.97 (29.44 - 30.5) | 30.01 (29.78 - 30.24) |
| **2020** | 36.65 (36.06 - 37.24) | 36.9 (36.65 - 37.16) |
| **Mean** | **25.51 (25.00 - 26.02)** | **28.89 (28.64 - 29.14)** |
| The data for urbanization is only available until 2020 in the CDC Wonder Database. | | |

**Supplemental Table 8. State-Stratified Age-Adjusted Mortality Rates per 100,000 Among U.S. Adults with Cardiac Arrest and Hypertension Listed on the Death Certificate, 2000–2024.**

| **Supplemental Table 8. State-Stratified Age-Adjusted Mortality Rates per 100,000 Among U.S. Adults with Cardiac Arrest and Hypertension Listed on the Death Certificate, 2000–2024.** | |
| --- | --- |
| **State** | **Age-Adjusted Rate (95% CI)** |
| Alabama | 29.53 (29.13–29.93) |
| Alaska | 9.78 (8.96–10.59) |
| Arizona | 21.57 (21.28–21.87) |
| Arkansas | 26.78 (26.3–27.26) |
| California | 67.04 (66.82–67.27) |
| Colorado | 12.78 (12.5–13.06) |
| Connecticut | 34.71 (34.24–35.18) |
| Delaware | 10.59 (10.06–11.13) |
| District of Columbia | 20.39 (19.4–21.37) |
| Florida | 21.72 (21.56–21.87) |
| Georgia | 46.53 (46.13–46.92) |
| Hawaii | 39.5 (38.68–40.32) |
| Idaho | 10.92 (10.48–11.37) |
| Illinois | 10.58 (10.43–10.73) |
| Indiana | 16.29 (16.03–16.55) |
| Iowa | 12.94 (12.64–13.25) |
| Kansas | 15.49 (15.13–15.86) |
| Kentucky | 15.94 (15.63–16.25) |
| Louisiana | 17.91 (17.58–18.24) |
| Maine | 9.36 (8.97–9.75) |
| Maryland | 8.52 (8.32–8.72) |
| Massachusetts | 20.52 (20.25–20.79) |
| Michigan | 12.45 (12.27–12.63) |
| Minnesota | 7.96 (7.76–8.15) |
| Mississippi | 91.96 (91.04–92.88) |
| Missouri | 14.17 (13.93–14.42) |
| Montana | 10.07 (9.58–10.55) |
| Nebraska | 30.86 (30.22–31.5) |
| Nevada | 33.85 (33.21–34.48) |
| New Hampshire | 15.96 (15.42–16.51) |
| New Jersey | 21.47 (21.22–21.71) |
| New Mexico | 14.76 (14.32–15.2) |
| New York | 62.27 (61.99–62.55) |
| North Carolina | 23.62 (23.36–23.88) |
| North Dakota | 18.91 (18.12–19.7) |
| Ohio | 30.29 (30.04–30.54) |
| Oklahoma | 25.93 (25.5–26.36) |
| Oregon | 9.34 (9.1–9.58) |
| Pennsylvania | 19.37 (19.18–19.55) |
| Rhode Island | 30.48 (29.68–31.28) |
| South Carolina | 27.33 (26.94–27.73) |
| South Dakota | 11.5 (10.93–12.08) |
| Tennessee | 23.27 (22.95–23.58) |
| Texas | 19.22 (19.06–19.38) |
| Utah | 11.05 (10.66–11.44) |
| Vermont | 13.07 (12.38–13.77) |
| Virginia | 15.21 (14.98–15.45) |
| Washington | 15.91 (15.65–16.16) |
| West Virginia | 30.67 (30.06–31.28) |
| Wisconsin | 8.27 (8.08–8.46) |
| Wyoming | 13.8 (12.97–14.62) |

**Supplemental Table 9 Cardiac Arrest and Hypertension related Forecast Age-Adjusted Mortality Rates, Stratified by Overall, Sex, Race, Age, Census, Urbanization in Adults in the United States, 2025 to 2035**

| **Supplemental Table 9, Cardiac Arrest and Hypertension related Forecast Age-Adjusted Mortality Rates, Stratified by Overall, Sex, Race, Age, Census, Urbanization in Adults in the United States, 2025 to 2035** | | | |
| --- | --- | --- | --- |
| **Stratification** | **Year** | **Forecast Value (LCI - UCI)** | **Model** |
| Female | 2025 | 26.39 (23.84–29.21) | ARIMA |
| Female | 2026 | 26.39 (22.86–30.21) | ARIMA |
| Female | 2027 | 26.39 (22.13–31.21) | ARIMA |
| Female | 2028 | 26.39 (21.54–32.21) | ARIMA |
| Female | 2029 | 26.39 (21.03–33.21) | ARIMA |
| Female | 2030 | 26.39 (20.58–33.21) | ARIMA |
| Female | 2031 | 26.39 (20.17–34.21) | ARIMA |
| Female | 2032 | 26.39 (19.80–35.21) | ARIMA |
| Female | 2033 | 26.39 (19.46–35.21) | ARIMA |
| Female | 2034 | 26.39 (19.14–36.21) | ARIMA |
| Female | 2035 | 26.39 (18.84–36.21) | ARIMA |
| Male | 2025 | 37.68 (33.78–42.04) | ARIMA |
| Male | 2026 | 37.68 (32.78–43.04) | ARIMA |
| Male | 2027 | 37.68 (31.78–45.04) | ARIMA |
| Male | 2028 | 37.68 (30.78–46.04) | ARIMA |
| Male | 2029 | 37.68 (29.78–48.04) | ARIMA |
| Male | 2030 | 37.68 (28.78–49.04) | ARIMA |
| Male | 2031 | 37.68 (28.78–50.04) | ARIMA |
| Male | 2032 | 37.68 (27.78–51.04) | ARIMA |
| Male | 2033 | 37.68 (27.78–52.04) | ARIMA |
| Male | 2034 | 37.68 (26.78–53.04) | ARIMA |
| Male | 2035 | 37.68 (26.78–54.04) | ARIMA |
| Overall | 2025 | 31.68 (28.53–35.18) | ARIMA |
| Overall | 2026 | 31.68 (27.53–36.18) | ARIMA |
| Overall | 2027 | 31.68 (26.53–37.18) | ARIMA |
| Overall | 2028 | 31.68 (25.53–39.18) | ARIMA |
| Overall | 2029 | 31.68 (25.53–40.18) | ARIMA |
| Overall | 2030 | 31.68 (24.53–40.18) | ARIMA |
| Overall | 2031 | 31.68 (24.53–41.18) | ARIMA |
| Overall | 2032 | 31.68 (23.53–42.18) | ARIMA |
| Overall | 2033 | 31.68 (23.53–43.18) | ARIMA |
| Overall | 2034 | 31.68 (22.53–44.18) | ARIMA |
| Overall | 2035 | 31.68 (22.53–44.18) | ARIMA |
| Hispanic | 2025 | 36.83 (31.92–42.50) | ARIMA |
| Hispanic | 2026 | 37.52 (31.39–44.84) | ARIMA |
| Hispanic | 2027 | 37.52 (31.39–44.84) | ARIMA |
| Hispanic | 2028 | 37.52 (31.39–44.84) | ARIMA |
| Hispanic | 2029 | 37.52 (31.39–44.84) | ARIMA |
| Hispanic | 2030 | 37.52 (31.39–44.84) | ARIMA |
| Hispanic | 2031 | 37.52 (31.39–44.84) | ARIMA |
| Hispanic | 2032 | 37.52 (31.39–44.84) | ARIMA |
| Hispanic | 2033 | 37.52 (31.39–44.84) | ARIMA |
| Hispanic | 2034 | 37.52 (31.39–44.84) | ARIMA |
| Hispanic | 2035 | 37.52 (31.39–44.84) | ARIMA |
| NH American Indian or Alaska Native | 2025 | 26.96 (20.70–35.10) | ARIMA |
| NH American Indian or Alaska Native | 2026 | 26.96 (18.55–39.16) | ARIMA |
| NH American Indian or Alaska Native | 2027 | 26.96 (17.06–42.59) | ARIMA |
| NH American Indian or Alaska Native | 2028 | 26.96 (15.89–45.71) | ARIMA |
| NH American Indian or Alaska Native | 2029 | 26.96 (14.93–48.65) | ARIMA |
| NH American Indian or Alaska Native | 2030 | 26.96 (14.11–51.47) | ARIMA |
| NH American Indian or Alaska Native | 2031 | 26.96 (13.40–54.21) | ARIMA |
| NH American Indian or Alaska Native | 2032 | 26.96 (12.77–56.89) | ARIMA |
| NH American Indian or Alaska Native | 2033 | 26.96 (12.20–59.53) | ARIMA |
| NH American Indian or Alaska Native | 2034 | 26.96 (11.69–62.14) | ARIMA |
| NH American Indian or Alaska Native | 2035 | 26.96 (11.22–64.72) | ARIMA |
| NH Asian or Pacific Islander | 2025 | 30.35 (26.16–35.20) | ARIMA |
| NH Asian or Pacific Islander | 2026 | 30.35 (24.60–37.44) | ARIMA |
| NH Asian or Pacific Islander | 2027 | 30.35 (23.46–39.25) | ARIMA |
| NH Asian or Pacific Islander | 2028 | 30.35 (22.55–40.84) | ARIMA |
| NH Asian or Pacific Islander | 2029 | 30.35 (21.77–42.30) | ARIMA |
| NH Asian or Pacific Islander | 2030 | 30.35 (21.09–43.66) | ARIMA |
| NH Asian or Pacific Islander | 2031 | 30.35 (20.48–44.95) | ARIMA |
| NH Asian or Pacific Islander | 2032 | 30.35 (19.93–46.19) | ARIMA |
| NH Asian or Pacific Islander | 2033 | 30.35 (19.43–47.38) | ARIMA |
| NH Asian or Pacific Islander | 2034 | 30.35 (18.97–48.54) | ARIMA |
| NH Asian or Pacific Islander | 2035 | 30.35 (18.54–49.66) | ARIMA |
| NH Black | 2025 | 58.07 (52.07–64.76) | ARIMA |
| NH Black | 2026 | 58.06 (50.56–66.69) | ARIMA |
| NH Black | 2027 | 58.06 (50.56–66.69) | ARIMA |
| NH Black | 2028 | 58.06 (50.56–66.69) | ARIMA |
| NH Black | 2029 | 58.06 (50.56–66.69) | ARIMA |
| NH Black | 2030 | 58.06 (50.56–66.69) | ARIMA |
| NH Black | 2031 | 58.06 (50.56–66.69) | ARIMA |
| NH Black | 2032 | 58.06 (50.56–66.69) | ARIMA |
| NH Black | 2033 | 58.06 (50.56–66.69) | ARIMA |
| NH Black | 2034 | 58.06 (50.56–66.69) | ARIMA |
| NH Black | 2035 | 58.06 (50.56–66.69) | ARIMA |
| NH White | 2025 | 27.98 (25.51–30.67) | ARIMA |
| NH White | 2026 | 27.98 (24.56–31.87) | ARIMA |
| NH White | 2027 | 27.98 (23.85–32.81) | ARIMA |
| NH White | 2028 | 27.98 (23.27–33.63) | ARIMA |
| NH White | 2029 | 27.98 (22.77–34.37) | ARIMA |
| NH White | 2030 | 27.98 (22.33–35.05) | ARIMA |
| NH White | 2031 | 27.98 (21.93–35.69) | ARIMA |
| NH White | 2032 | 27.98 (21.56–36.30) | ARIMA |
| NH White | 2033 | 27.98 (21.22–36.88) | ARIMA |
| NH White | 2034 | 27.98 (20.91–37.43) | ARIMA |
| NH White | 2035 | 27.98 (20.61–37.97) | ARIMA |
| Northeast | 2025 | 34.65 (30.87–38.90) | ARIMA |
| Northeast | 2026 | 34.83 (30.87–39.90) | ARIMA |
| Northeast | 2027 | 34.93 (30.87–40.90) | ARIMA |
| Northeast | 2028 | 34.99 (30.87–40.90) | ARIMA |
| Northeast | 2029 | 35.02 (30.87–40.90) | ARIMA |
| Northeast | 2030 | 35.03 (30.87–40.90) | ARIMA |
| Northeast | 2031 | 35.04 (30.87–40.90) | ARIMA |
| Northeast | 2032 | 35.05 (30.87–40.90) | ARIMA |
| Northeast | 2033 | 35.05 (30.87–40.90) | ARIMA |
| Northeast | 2034 | 35.05 (30.87–40.90) | ARIMA |
| Northeast | 2035 | 35.05 (30.87–40.90) | ARIMA |
| Midwest | 2025 | 20.22 (18.87–22.90) | ARIMA |
| Midwest | 2026 | 20.22 (17.87–23.90) | ARIMA |
| Midwest | 2027 | 20.22 (16.87–24.90) | ARIMA |
| Midwest | 2028 | 20.22 (16.87–25.90) | ARIMA |
| Midwest | 2029 | 20.22 (15.87–25.90) | ARIMA |
| Midwest | 2030 | 20.22 (15.87–26.90) | ARIMA |
| Midwest | 2031 | 20.22 (15.87–26.90) | ARIMA |
| Midwest | 2032 | 20.22 (14.87–27.90) | ARIMA |
| Midwest | 2033 | 20.22 (14.87–27.90) | ARIMA |
| Midwest | 2034 | 20.22 (14.87–28.90) | ARIMA |
| Midwest | 2035 | 20.22 (14.87–28.90) | ARIMA |
| South | 2025 | 27.18 (24.87–30.90) | ARIMA |
| South | 2026 | 27.18 (23.87–31.90) | ARIMA |
| South | 2027 | 27.18 (22.87–33.90) | ARIMA |
| South | 2028 | 27.18 (21.87–34.90) | ARIMA |
| South | 2029 | 27.18 (21.87–35.90) | ARIMA |
| South | 2030 | 27.18 (20.87–36.90) | ARIMA |
| South | 2031 | 27.18 (20.87–36.90) | ARIMA |
| South | 2032 | 27.18 (19.87–37.90) | ARIMA |
| South | 2033 | 27.18 (19.87–38.90) | ARIMA |
| South | 2034 | 27.18 (18.87–39.90) | ARIMA |
| South | 2035 | 27.18 (18.87–39.90) | ARIMA |
| West | 2025 | 47.28 (42.87–52.90) | ARIMA |
| West | 2026 | 47.28 (40.87–55.90) | ARIMA |
| West | 2027 | 47.28 (39.87–56.90) | ARIMA |
| West | 2028 | 47.28 (38.87–58.90) | ARIMA |
| West | 2029 | 47.28 (37.87–60.90) | ARIMA |
| West | 2030 | 47.28 (36.87–61.90) | ARIMA |
| West | 2031 | 47.28 (35.87–62.90) | ARIMA |
| West | 2032 | 47.28 (34.87–64.90) | ARIMA |
| West | 2033 | 47.28 (34.87–65.90) | ARIMA |
| West | 2034 | 47.28 (33.87–66.90) | ARIMA |
| West | 2035 | 47.28 (33.87–67.90) | ARIMA |
| Younger Adults (25-44) | 2025 | 1.75 (1.44–2.10) | ARIMA |
| Younger Adults (25-44) | 2026 | 1.75 (1.34–2.26) | ARIMA |
| Younger Adults (25-44) | 2027 | 1.75 (1.24–2.38) | ARIMA |
| Younger Adults (25-44) | 2028 | 1.75 (1.14–2.49) | ARIMA |
| Younger Adults (25-44) | 2029 | 1.75 (1.14–2.60) | ARIMA |
| Younger Adults (25-44) | 2030 | 1.75 (1.04–2.69) | ARIMA |
| Younger Adults (25-44) | 2031 | 1.75 (0.94–2.78) | ARIMA |
| Younger Adults (25-44) | 2032 | 1.75 (0.94–2.86) | ARIMA |
| Younger Adults (25-44) | 2033 | 1.75 (0.94–2.94) | ARIMA |
| Younger Adults (25-44) | 2034 | 1.75 (0.84–3.02) | ARIMA |
| Younger Adults (25-44) | 2035 | 1.75 (0.84–3.10) | ARIMA |
| Middle-Aged Adults (45-64) | 2025 | 16.04 (13.4–18.4) | ARIMA |
| Middle-Aged Adults (45-64) | 2026 | 16.04 (13.4–19.5) | ARIMA |
| Middle-Aged Adults (45-64) | 2027 | 16.04 (12.4–20.4) | ARIMA |
| Middle-Aged Adults (45-64) | 2028 | 16.04 (12.4–21.1) | ARIMA |
| Middle-Aged Adults (45-64) | 2029 | 16.04 (11.4–21.8) | ARIMA |
| Middle-Aged Adults (45-64) | 2030 | 16.04 (11.4–22.5) | ARIMA |
| Middle-Aged Adults (45-64) | 2031 | 16.04 (11.4–23.1) | ARIMA |
| Middle-Aged Adults (45-64) | 2032 | 16.04 (10.4–23.7) | ARIMA |
| Middle-Aged Adults (45-64) | 2033 | 16.04 (10.4–24.3) | ARIMA |
| Middle-Aged Adults (45-64) | 2034 | 16.04 (10.4–24.8) | ARIMA |
| Middle-Aged Adults (45-64) | 2035 | 16.04 (9.94–25.3) | ARIMA |
| Older Adults (65+) | 2025 | 129.79 (1174–142) | ARIMA |
| Older Adults (65+) | 2026 | 129.79 (1134–148) | ARIMA |
| Older Adults (65+) | 2027 | 129.79 (1094–153) | ARIMA |
| Older Adults (65+) | 2028 | 129.79 (1074–157) | ARIMA |
| Older Adults (65+) | 2029 | 129.79 (1044–160) | ARIMA |
| Older Adults (65+) | 2030 | 129.79 (1024–164) | ARIMA |
| Older Adults (65+) | 2031 | 129.79 (1004–167) | ARIMA |
| Older Adults (65+) | 2032 | 129.79 (98.4–170) | ARIMA |
| Older Adults (65+) | 2033 | 129.79 (97.4–173) | ARIMA |
| Older Adults (65+) | 2034 | 129.79 (95.4–175) | ARIMA |
| Older Adults (65+) | 2035 | 129.79 (94.4–178) | ARIMA |
| Urban | 2021 | 34.10 (32.85–35.43) | Prophet |
| Urban | 2022 | 34.60 (33.32–35.32) | Prophet |
| Urban | 2023 | 35.65 (34.30–37.87) | Prophet |
| Urban | 2024 | 37.31 (35.80–38.65) | Prophet |
| Urban | 2025 | 37.87 (36.21–39.83) | Prophet |
| Urban | 2026 | 38.43 (36.63–40.52) | Prophet |
| Urban | 2027 | 39.60 (37.62–41.28) | Prophet |
| Urban | 2028 | 41.44 (39.14–43.61) | Prophet |
| Urban | 2029 | 42.06 (39.38–44.94) | Prophet |
| Urban | 2030 | 42.68 (39.69–45.03) | Prophet |
| Urban | 2031 | 43.98 (40.47–47.79) | Prophet |
| Urban | 2032 | 46.03 (42.08–50.59) | Prophet |
| Urban | 2033 | 46.72 (42.30–51.85) | Prophet |
| Urban | 2034 | 47.40 (42.60–52.94) | Prophet |
| Urban | 2035 | 48.85 (43.08–54.30) | Prophet |
| Rural | 2021 | 35.44 (34.34–36.60) | Prophet |
| Rural | 2022 | 36.20 (35.05–37.66) | Prophet |
| Rural | 2023 | 37.87 (36.63–39.87) | Prophet |
| Rural | 2024 | 40.59 (39.01–42.39) | Prophet |
| Rural | 2025 | 42.14 (40.31–44.76) | Prophet |
| Rural | 2026 | 43.05 (40.63–45.32) | Prophet |
| Rural | 2027 | 45.04 (42.03–47.22) | Prophet |
| Rural | 2028 | 48.27 (44.64–52.36) | Prophet |
| Rural | 2029 | 50.12 (45.65–54.76) | Prophet |
| Rural | 2030 | 51.20 (45.85–56.07) | Prophet |
| Rural | 2031 | 53.57 (47.44–60.27) | Prophet |
| Rural | 2032 | 57.40 (49.94–65.75) | Prophet |
| Rural | 2033 | 59.60 (50.75–69.63) | Prophet |
| Rural | 2034 | 60.89 (51.02–71.13) | Prophet |
| Rural | 2035 | 63.71 (52.30–76.24) | Prophet |
| LCI = Lower Confidence Interval |  |  |  |
| UCI = Upper confidence Interval |  |  |  |
